# CREB1 Facilitates GABAergic Neural Differentiation of Human Mesenchymal Stem Cells through BRN2 for Pain Alleviation and Locomotion Recovery after Spinal Cord Injury

**DOI:** 10.3390/cells13010067

**Published:** 2023-12-28

**Authors:** Yanbing Kao, Hanming Zhu, Yu Yang, Wenyuan Shen, Wei Song, Renjie Zhang, Yanchun Liu, Haoyun Liu, Xiaohong Kong

**Affiliations:** 1 Orthopedic Research Center of Qilu Hospital, Shandong University, Jinan 250100, China; 2 Advanced Medical Research Institute, Cheeloo College of Medicine, Shandong University, Jinan 250100, China

**Keywords:** CREB1, GABAergic neuron cells, spinal cord injury

## Abstract

The transplantation of GABAergic neuron cells has been reported to alleviate nerve pain and improve motor function after spinal cord injury (SCI). However, human mesenchymal stem cell (hMSC) differentiation into GABAergic neuron cells in a sufficient quantity remains to be accomplished. From a database screening, cAMP-responsive element-binding protein 1 (CREB1) was chosen as a potential modulator due to its critical role in the protein–protein interaction of genes related to GABAergic neural differentiation. Here, CREB1 was overexpressed in transfected hMSCs, where CREB1 could induce differentiation into GABAergic neuron cells with an upregulation of Map2 and GAD1 by 2- and 3.4-fold, respectively. Additionally, GABAergic neural differentiation was enhanced, while Notch signaling was inhibited, and BRN2 transcriptional activation played an important role in neuronal maturation. Moreover, transfected hMSCs injected into immunocompromised mice caused by CsA exhibited the neuronal markers Tuj1 and Map2 via the intraspinal route, suggesting an improvement in survival and neural differentiation. Significantly, improvement in both BMS scores (6.2 ± 1.30 vs. 4 ± 0) and thermal hyperalgesia latency (7.74 ± 2.36 s vs. 4.52 ± 0.39 s) was seen compared with the SCI naïve treatment at 4 weeks post-transplantation. Our study demonstrates that CREB1 is crucial in generating induced GABAergic neuron cells (iGNs) originating from hMSCs. Transplanting iGNs to injured spinal cord provides a promising strategy for alleviating neuropathic pain and locomotion recovery after SCI.

## 1. Introduction

SCI is a severe neurological disease that can lead to permanent neurological impairments and several neurological complications [1,2]. Although treatment options are available for SCI patients, such as surgical decompression to prevent secondary damage and rehabilitation to activate residual neural circuitry and restore connections, they are unable to achieve significant neurological recovery due to the limited regenerative capacity of spinal cord neurons and the presence of inhibitory factors at the lesion site [3,4]. The long-term care and treatment costs of SCI patients have been a major burden on society [5].

In the past ten years, cell-based therapy has emerged as a potential strategy for SCI repair. Various cell types have been developed for treatments of SCI, including Schwann cells [6], neural stem cells (NSCs), neural progenitor cells (NPCs) [7,8,9], oligodendrocyte precursor cells (OPCs) [10], olfactory ensheathing cells (OECs) [11], and mesenchymal stem cells (MSCs) [12]. Despite the encouraging outcomes of these cells in restoring motor function, exerting therapeutic effects by providing neuroprotection, inhibiting inflammation, and replacing lost neurons at the lesion site, they do not exhibit significant analgesic effects. As we know, the decrease in central inhibition caused by the impairment of inhibitory interneurons such as GABAergic neurons after SCI is a major pathological component of neuropathic pain [13]. Thus, research studies have attempted the injection of GABAergic neuron cells into injured spinal cord to alleviate neuropathic pain, which showed promising effects on SCI mouse models. Recent publications reported that transplantation of GABAergic neuron cells or GABAergic progenitor cells indirectly improved motor function by alleviating neuropathic pain and muscle spasticity after SCI [14,15].

GABAergic neuron cells for transplantation are derived from GABAergic progenitor cells originating from the embryonic medial ganglionic eminence (MGE) and embryonic stem cell-derived neural precursor cells (ESC-NPCs) [16,17]. Extracting GABAergic neuron cells from human embryonic tissues leads to some ethical and safety concerns regarding multilineage differentiation potential, and thus, researchers have tried to convert induced pluripotent stem cells (iPSC) into GABAergic neuron cells, where they have made great progress [15,18]. However, the clinical application of these methods is limited due to their complicated technical manipulation, such as gene editing and cellular reprogramming, and potential tumorigenicity [19].

MSCs are a kind of adult stem cell derived from mesenchymal tissues. Human MSCs (hMSCs) can be obtained from various sources, including the umbilical cord, bone marrow, adipose tissue, and even dental pulp [20,21,22]. In this study, we utilized umbilical cord-derived MSCs. They have great advantages such as easy accessibility, easy manipulation, and multipotency. Previous studies have focused on the paracrine effects of MSCs and paid little attention to the potential of hMSC differentiation into GABAergic neuron cells. The goal of this study was to determine whether CREB1 facilitates GABAergic neural differentiation of hMSCs. Here, we report that CREB1, a transcription factor associated with neuroregeneration and chosen from an open database screening, may help alleviate neuropathic pain for SCI recovery. Moreover, hMSCs exhibited neuronal marker expression. Map2 and GAD1 expression was enhanced upon the upregulation of constitutively active cAMP-responsive element-binding protein (CA-CREB). Consistent with the protein expression level, RNA-seq showed significant upregulation of key genes, including Snap25 and Dnm3, involved in the synaptic vesicle cycle. Additionally, SCI mice exhibited pain relief and improved locomotion at 4 weeks after the injection of these iGNs to injured spinal cord.

## 2. Methods

### 2.1. Acquisition and Characterization of Mesenchymal Stem Cells

hUC-MSCs of P2 were obtained from Shandong Qilu Stem Cells Engineering Company and were maintained and expanded in α-MEM basal medium (VivaCell, Shanghai, China), which contained 10% fetal bovine serum (FBS, VivaCell, Shanghai, China). Immunophenotypic markers (CD34, CD45, CD73, CD90, and CD105) were detected by means of flow cytometry. The differentiation potential of MSCs was assessed using an Induced Differentiation Kit (HUXUC-90021, HUXUC-90031, HUXUC-90041, Oricell, Guangzhou, China).

### 2.2. Generation of iPSC-Derived GABA Neurons and iGNs

iPSC-derived GABA neuron cells are GABAergic neuron cells originating from iPSCs. iPSCs are from hiPSCs (ATCC, ACS1030). iPSCs were induced into GABAergic neurons via chemical induction such as IWP2 and SAG. In this study, we analyzed two publicly open datasets (iPSC-derived GABAergic neuron versus iPSCs) obtained from the Gene Expression Omnibus (GEO) database (https://www.ncbi.nlm.nih.gov/geo/ (accessed on 26 September 2021)). The GEO series ID used for searching the GEO website is GSE115565 (the link to the datasets is https://www.ncbi.nlm.nih.gov/geo/query/acc.cgi?acc=GSE115565 (accessed on 26 September 2021)) [18]. iGNs were induced GABAergic neuron cells originating from hUC-MSCs. CA-CREB was overexpressed in hUC-MSCs over 2-week culturing to induce iGNs.

### 2.3. The Lentivirus Package and Cell Transfection

The plasmid for lentivirus packaging was obtained from Hanbio Tech (Shanghai, China). The sequence encoding the fusion protein between HSV VP16 (aa 363 to 490) and CREB (aa 88. to 341) was constructed on a pHBLV backbone plasmid. Lentiviral packaging plasmids (pMD2.G and psPAX2) were co-transfected with the pHBLV backbone plasmid into HEK293T cells for virus production. Viral supernatants were collected 48 h and 72 h after transfection, centrifuged at 72,000× *g* for 2 h, resuspended, and filtered through 0.45 μm filters (Millipore, Burlington, MA, USA). hUC-MSCs cells were plated on 24-well plate and transfected with lentivirus with MOI = 10. These cells were maintained in the virus-containing medium for 8 h and washed, and the medium was replaced.

### 2.4. EdU Assay

The EdU assay was conducted using a kit (RiboBio, Guangzhou, China). Cells were exposed to a 50 μM EdU solution at 37 °C for 3 h. Afterward, the cells were fixed with 4% paraformaldehyde for 30 min at room temperature. Subsequently, cells were permeabilized with PBS containing 0.5% Triton X-100 for 20 min, followed by three washes with PBS. Cells were then stained with Apollo staining reagent for 20 min and washed three times with PBS for 5 min each. Finally, cell nuclei were stained with Hoechst and observed using a fluorescence microscope.

### 2.5. RNA-seq

TRIzol reagent (15596026, Invitrogen, Waltham, MA, USA) was used to extract total RNA from 7-day MSCs that were transfected with CA-CREB lentivirus, following the manufacturer’s instructions. Novogene (Beijing, China) conducted RNA-sequencing for the obtained RNA samples. The NEBNext Ultra™ Directional RNA Library Prep Kit for Illumina (New England Biolabs, Ipswich, MA, USA) was used to generate sequencing libraries. An index of the reference genome was built using Hisat2 (v2.0.5) and paired-end clean reads were aligned to the reference genome using Hisat2 (v2.0.5). Following the alignment, feature counts (v1.5.0-p3) were used to analyze differential gene expression, utilizing the BAM files obtained from each individual alignment.

### 2.6. Reverse Transcription and Quantitative PCR

Total RNA was extracted using TRIzol following manufacturer’s instructions (15596026, Invitrogen, Waltham, MA, USA). cDNA was synthesized with a cDNA reverse transcription kit (KR116-02, Tiangen, China) from 2 μg total RNA. The qPCR was performed using SYBR Green (AQ601, TransGen Biotech, Beijing, China) and the LC96 real-time PCR detection system (Roche, Mannheim, Germany) according to provided instructions.

### 2.7. Western Blotting

Cells were lysed using RIPA buffer containing a protease inhibitor (B14001, Bimake, Houston, TX, USA) following manufacturer’s instructions. Protein concentration was determined using the BCA Protein Assay Kit (Beyotime Biotechnology, Shanghai, China). A total of 20 μg of protein was separated using 10% sodium dodecyl sulfate polyacrylamide gel electrophoresis and transferred to a polyvinylidene difluoride membrane (0.25 μm; Millipore, Burlington, MA, USA). Membranes were blocked with 5% BSA (Beyotime Biotechnology, Shanghai, China) for 1 h, followed by incubation with primary antibodies overnight at 4 °C on a horizontal shaker. The next day, a secondary antibody (1:10,000; Proteintech, Wuhan, China) was applied for 1 h at room temperature. The primary antibodies used were mouse anti-GAPDH (1:10,000; HRP-60004, Proteintech, Wuhan, China), rabbit anti-Tuj1 (1:1000; ab18207, Abcam, Waltham, MA, USA), rabbit anti-Map2 (1:1000; ab32454, Abcam, Waltham, MA, USA), rabbit anti-BRN2 (1:1000; 12137, CST, Danvers, MA, USA), and rabbit anti-Synapsin 1 (1:1000; ab32127, Abcam, Waltham, MA, USA). Protein bands were visualized using an enhanced chemiluminescence kit (Bio-Rad, Hercules, CA, USA), and grayscale analysis of the Western blot images was performed using ImageJ.

### 2.8. Chromatin Immunoprecipitation (ChIP)

The ChIP Assay Kit (P2078, Beyotime Biotechnology, Shanghai, China) was used for conducting ChIP assays, following the manufacturer’s instructions. The cells were fixed with 1% paraformaldehyde for 10 min, followed by the addition of 0.125 M glycine to halt DNA–protein crosslinking at room temperature for 5 min. To generate chromatin fragments, the cells were treated with SDS lysis buffer containing protease inhibitors and subjected to ultrasonic fragmentation. A portion of the generated fragments was designated as “Input.” The remaining lysates were incubated overnight at 4 °C with Protein G magnetic beads and mouse Flag antibody (4 μg/1 mg protein; F3165, Sigma-Aldrich, St. Louis, MO, USA) to form a DNA–antibody–magnetic beads complex. After elution and purification, the DNA was labeled “ChIP”. As a negative control, mouse IgG (4 μg/1 mg protein; 12-371, Millipore, Darmstadt, Germany) was employed. The final purified DNA fragments were analyzed using RT-qPCR.

### 2.9. Plasmid Construction and Dual-Luciferase Reporter Assay

A 1510-bp human BRN2 promoter was inserted in the pGL-3 basic vector. The constructed plasmid was named pGL_1510_-Luc. Further deletion of the pGL_1510_-Luc generated pGL_1410_-Luc and pGL_320_-Luc reporters containing 1410 bp, 320 bp of BRN2 promoter, respectively. The 293T cells were co-transfected with pGL3 plasmids containing truncated mutants of BRN2 promoter, Renilla luciferase plasmid pRL-TK and plasmid overexpressing CA-CREB (pHBLV-CREB) or vector (pHBLV). After 48 h, the luciferase activity assay was performed using the Dual Luciferase Reporter Assay Kit (RG088S, Beyotime Biotechnology, Shanghai, China) following provided instruction. Finally, the fluorescence intensity was detected using a microplate reader (Thermo Scientific, Waltham, MA, USA).

### 2.10. Preparation of Transplanted Cell

In this study, we chose 3-day MSCs transfected with CA-CREB lentivirus for transplantation. On the transplantation day, the cells were collected from the culture plates using 0.25% trypsin/EDTA, followed by washing and resuspension in cold PBS. The concentration of the cells in the resuspended solution for transplantation was 2 × 10^5^ cells/μL. The cell suspension was kept on ice until the transplantation procedure.

### 2.11. Contusion SCI Model and Cell Transplantation

All animal experiments were conducted in accordance with the guidelines set by the Ethics Committee of Qilu Hospital, Shandong University (KYLL-2021(KS)-982). Twenty-six female C57BL/6 mice (22–25 g) of 8–10 weeks of age were obtained from Vital River Laboratory Animal Technology Co., Ltd. The mice were randomly divided into four groups (sham *n* = 5, PBS *n* = 5, hUC-MSC *n* = 8, and iGN group *n* = 8). On the day before modeling, all mice were administered cyclosporine A (HY-B0579, MCE, Middlesex, NJ, USA) via intraperitoneal injection at a dosage of 10 mg/kg/day. The contusion SCI model was established on the next day following previously published protocols [23,24]. After SCI, the treatment was applied to the mice in different groups. In the experimental hUC-MSC and iGN groups, a 3 μL suspension of transplanted cells (2 × 10^5^ cells/μL) was injected to lesion site immediately after SCI, as previously described [25]. In the PBS group, the mice received 3 μL PBS injection using a similar procedure. The sham group was treatment naive. The mice were housed in an appropriate environment with access to ample food and water, and the temperature was maintained at 20–25 °C. Manual bladder emptying was performed twice daily. All mice were administered cyclosporine A (HY-B0579, MCE, Middlesex, NJ, USA) via intraperitoneal injection at a dosage of 10 mg/kg/day continuing until the end of the experiment. At 2 weeks post-transplantation, 3 mice from both hUC-MSC and iGN groups were euthanized, and their spinal cord tissues collected to assess the survival and integration of the transplanted cells.

### 2.12. Perfusion and Sectioning

Mice were anesthetized with isoflurane, and the heart was exposed after opening the chest cavity. Precooled PBS was then perfused through the heart until no blood was observed flowing out and the liver color paled. Subsequently, pre-cooled 4% paraformaldehyde was transcardially perfused. Bilateral lamina was carefully removed using spring scissors to fully expose the spinal cord. The spinal cord was collected, and then fixed in 4% paraformaldehyde for 24 h. To prepare the tissue for further analysis, a concentration gradient sucrose solution was used for dehydration. Subsequently, the dehydrated spinal cords were embedded in OCT mounting media and sectioned at a thickness of 8 μm using a cryotome (Leica, CM3050S, Wetzlar, Germany).

### 2.13. Immunofluorescence Staining

Cultured cells or spinal cord tissues were fixed with 4% paraformaldehyde, and then placed in blocking buffer (20% normal goat plasma, 10% FBS and 0.25% Triton X-100 in PBS) for 1 h. Following blocking, the sections were exposed to primary antibodies and incubated overnight at 4 °C. The primary antibodies used were rabbit anti-Tuj1 (1:1000; ab18207, Abcam, Waltham, MA, USA), rabbit anti-Map2 (1:1000; ab183830, Abcam, Waltham, MA, USA), mouse anti-GAD1 (1:200; 67648-1-Ig, Proteintech, Wuhan, China), rabbit anti-Synapsin 1 (1:1000; ab32127, Abcam, Waltham, MA, USA), and mouse anti-NeuN (1:1000; ab104224, Abcam, Waltham, MA, USA). On the subsequent day, the sections underwent three washes with PBS for 5 min each, followed by incubation with the corresponding secondary antibody conjugated to Alexa Fluor 488, 555, or 647 (1:1000; A32723 A32727, Invitrogen, Waltham, MA, USA; ab150077 ab150078 ab150115, Abcam, Waltham, MA, USA) for 1.5 h at room temperature. The fluorescence intensity was analyzed using a laser microscope (Zeiss LSM 980, Jena, Germany).

### 2.14. Behavioral Analysis

Basso mouse scale (BMS): To assess hindlimb locomotor behavior recovery in mice, BMS scores were conducted at various time points: prior to SCI; on day 1, 4, and 7 post-SCI; and then weekly for 4 weeks, following the Basso mouse scale guidelines [26]. Before the tests, mice were acclimated to the round open field environment with a diameter of 1 m. Transparent glass panels surrounded the field, enabling observers to evaluate hindlimb locomotor behavior. Two blinded investigators carried out the experiments, randomly testing and scoring mice from different experimental groups. Each mouse was individually placed in the open field and observed for 4 min. BMS scores ranged from 0 to 9, with each score representing a distinct degree of hindlimb locomotor behavior.

Catwalk gait analysis: The CatWalk XT system (Noldus, Wageningen, The Netherlands) was used to assess the recovery of lower limb motor function in mice, as previously described [24]. CatWalk testing was conducted in a dark room, and a traversal of the runway by the mice within 15 s was considered a valid footprint recording. The collected mouse footprint data were analyzed using CatWalk XT software (latest v. 10.6). The experimenters conducting the tests were blinded to the treatment groups.

Von Frey and hot plate assays: From one week before the surgery, the mice were exposed to the testing environment to familiarize them with the surroundings. They spent 30 min in the environment before undergoing mechanical and thermal tests. Following SCI, the plantar surface of both ipsilateral and contralateral hind paws was stimulated using electrical von Frey tips (BIO-EVF5, Bioseb, Vitrolles, France) to measure mechanical thresholds. The maximum force (in grams) at which withdraw response was observed was automatically recorded. Each mouse underwent three tests, with a 15 s interval between each test. For the hot plate assay, a hot plate device (BIO CHP, Bioseb, Vitrolles, France) was utilized. The temperature was set at 55 °C, and the maximum exposure time was set at 60 s to prevent tissue damage. The thermal hyperalgesia latency was recorded. The experimenters conducting the tests were blinded to the treatment groups.

### 2.15. Statistical Analysis

Statistical analyses were conducted using GraphPad Prism 8 (GraphPad Software, San Diego, CA, USA). Results were analyzed using one-way ANOVA with Holm–Sidak test for multiple comparisons or Student’s *t*-test for pairwise comparisons. Statistical significance was defined as a *p*-value less than 0.05.

## 3. Results

### 3.1. The Characterization of hUC-MSCs

**Figure 1:**
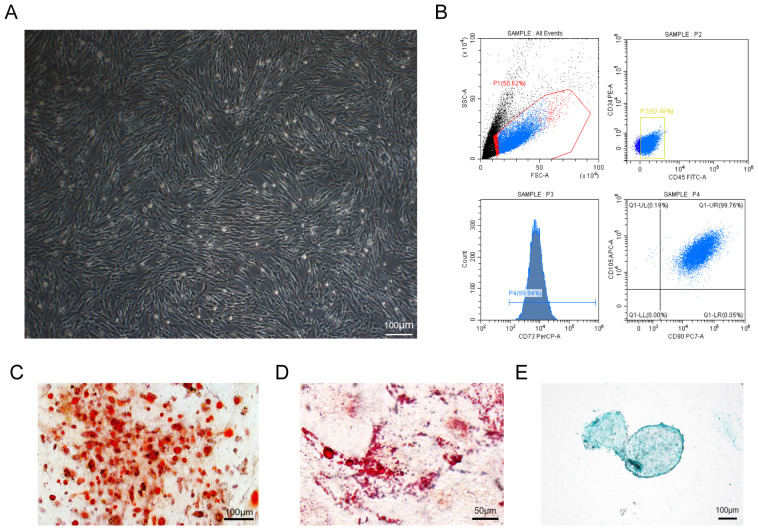
Characterization of hUC-MSC differentiation potential. (**A**) Morphology of cultured MSCs under an inverted phase-contrast microscope. Scale bars: 100 μm. (**B**) Identification of surface markers of CD34, CD45, CD73, CD90, and CD105 in MSCs using flow cytometry. (**C**) Capacity of osteogenic differentiation using alizarin red staining. Scale bars: 100 μm. (**D**) Capacity of adipogenic differentiation using oil red staining. Scale bars: 50 μm. (**E**) Chondrogenic differentiation capacity using Alcian blue staining. Scale bars: 100 μm.

The isolated hUC-MSCs exhibited spindle-like cells at 3 days after passage and reached 80–90% confluence (Figure 1A). The cellular surface markers CD73, CD90, and CD105 were identified positively as 99.94%, 99.81%, and 99.95%, respectively, using flow cytometry analysis in hUC-MSCs, where CD34 or CD45 of the hematopoietic stem cells markers were undetectable (Figure 1B). Subsequently, the osteogenic, adipogenic, and chondrogenic differentiation potential of hUC-MSCs was detected. Cultured hUC-MSCs were stained red by using alizarin red or oil red throughout the 2-week culture in the induced medium, indicating their differentiation into osteoblasts and adipocytes (Figure 1C,D). Additionally, chondrogenic pellets generated from the suspension culture of hUC-MSCs up to 4 weeks were stained positive for cartilage formation by using Alcian blue (Figure 1E). As expected, the hUC-MSCs used in this study showed trilineage differentiation ability.

### 3.2. Ectopic Expression of CA-CREB Induced MSC to Neuronal Phenotype In Vitro

**Figure 2:**
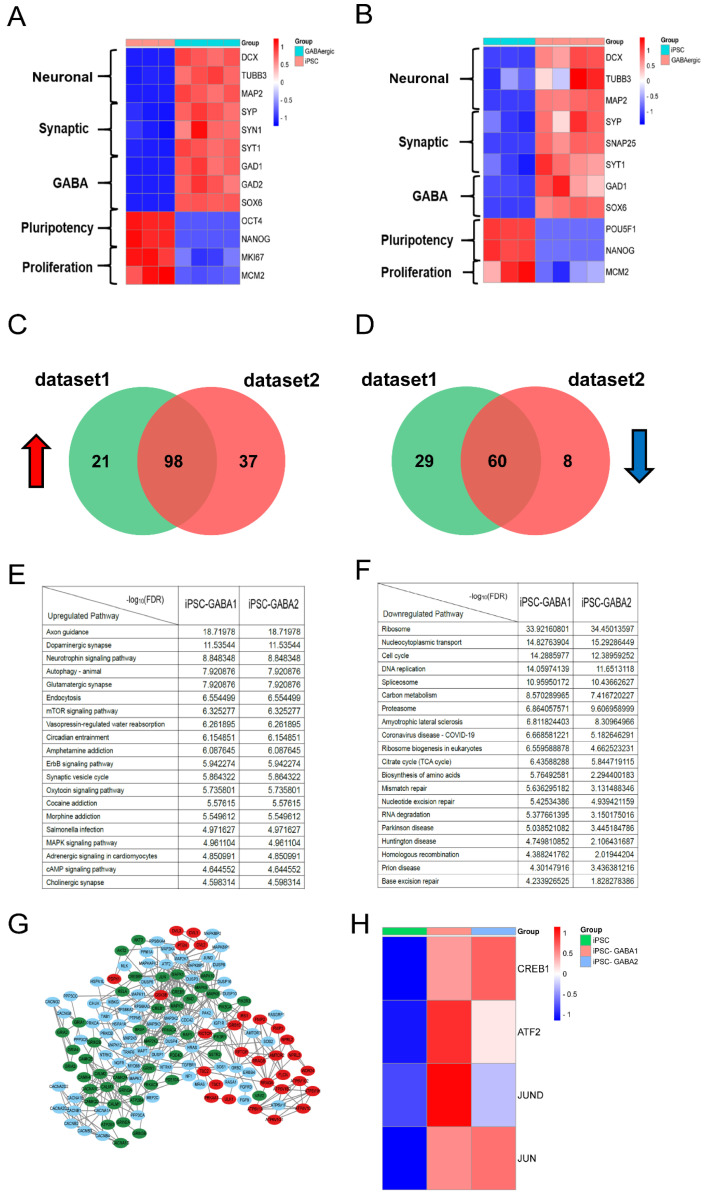
Identification of key pathways and regulators in GABAergic neural differentiation. The RNA-seq datasets (iPSCs versus iPSC-derived GABAergic neuron cells) were collected from the GEO database (GSE115565) and then the gene files were standardized to perform KEGG (ClusterProfiler, Version 3.8.1) and PPI analysis (*STRING* http://string-db.org/). (**A**) The heatmap of GABAergic neuronal marker genes in dataset 1(GSM3182508-GSM3182511). (**B**) The heatmap of GABAergic neuronal markers in dataset 2(GSM3182512-GSM3182515). (**C**) Venn diagram of 98 overlapping upregulated signaling pathways in both RNA-seq datasets, 21 unique upregulated pathways in dataset1 and 37 unique upregulated pathways in dataset 2 (*p* < 0.05). (**D**) Venn diagram of 60 overlapping downregulated signaling pathways in both RNA-seq datasets, 29 unique downregulated pathways in dataset1 and 8 unique downregulated pathways in dataset 2 (*p* < 0.05). (**E**) The top 20 upregulated pathways with lowest FDR shared between two RNA-seq datasets. (**F**) The top 20 downregulated pathways with lowest FDR shared between two RNA-seq datasets. (**G**) Protein–protein interaction of DEGs (FDR < 0.05) involved in MAPK (blue points), mTOR (red points), and cAMP pathways (green points) in iPSC-derived GABAergic neuron cells. (**H**) The heatmap of CREB1 and three other related TFs (ATF2, JUND, and JUN) in iPSCs and iPSC-derived GABAergic neuron cells.

To explore whether CREB1 is the key regulator in GABAergic neural differentiation, we evaluated the expression of GABAergic neuronal markers in two available RNA-seq datasets comparing iPSCs with iPSC-derived GABAergic neuron cells. As shown in Figure 2A,B, the expression of the GABAergic neuronal marker including Map2, GAD1, and Syp was significantly enhanced in iPSC-derived GABAergic neuron cells compared with iPSCs. Subsequently, significant differentially expressed genes were analyzed using Kyoto Encyclopedia of Genes and Genomes (KEGG) analysis to investigate the pathways upregulated or downregulated in those two datasets. The Venn diagrams showed that despite differentials in the level of expression, there were 98 upregulated and 60 downregulated signaling pathways shared between two datasets (Figure 2C,D). The top 20 upregulated or downregulated pathways out of the shared pathways with lowest FDR are listed in Figure 2E,F. Our interest is in neural differentiation and development-related pathways from the identified shared pathways, which include the mTOR, MAPK, and cAMP pathways. To further investigate the interactions between proteins involved in these pathways, we constructed a protein–protein interaction (PPI) network. Notably, we observed strong interactions between CREB1 associated with neural development and other proteins like CREBBP and PRKACA in the PPI network (Figure 2G). Additionally, the heatmap revealed that the expression of CREB1 and CREB1-related transcription factors (TFs) such as Jun, JunD, and ATF2 was significantly upregulated in iPSC-derived GABAergic neuron cells compared with iPSCs (Figure 2H), suggesting CREB1 is a key transcriptional factor that could drive the hMCSc differentiation to GABAergic neuron cells.

**Figure 3:**
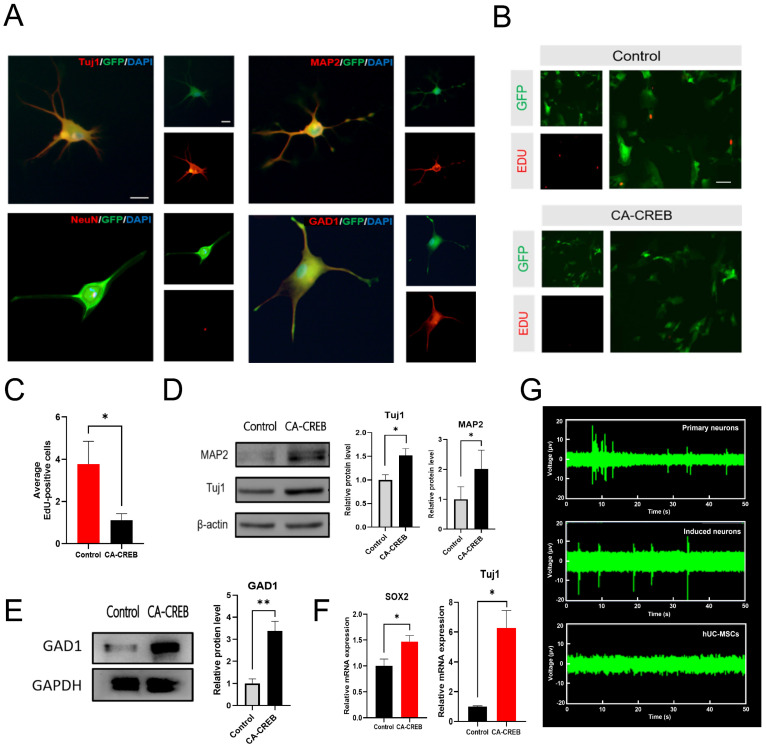
Ectopic expression of CA-CREB induces MSCs to neuronal phenotypes. (**A**) Representative images showing Tuj1, Map2, NeuN, and GAD1 immunostaining (GABAergic neuronal markers) in hUC-MSCs at 14 days after transfection with lentivirus overexpressing CA-CREB. Scale bars, 25 μm. (**B**,**C**) Representative images of EdU staining of hUC-MSCs at 4 days after transfection with lentivirus overexpressing CA-CREB or GFP (as a control) and quantitative analysis. Scale bars, 25 μm. (**D**) Representative Western blot showing the expression of Tuj1 and Map2 at 7 days after transfection with lentivirus overexpressing CA-CREB or GFP (as a control) and quantitative analysis. (**E**) Representative Western blot showing the expression of GAD1 at 7 days after transfection with lentivirus overexpressing CA-CREB or GFP (as a control) and quantitative analysis. (**F**) RNA expression of Sox2 and Tuj1 detected using RT-qPCR at 2 days after transfection with lentivirus overexpressing CA-CREB or GFP (as a control). Data are presented as mean ± SEM. Results were analyzed using *t*-test. Statistical significance: * *p* < 0.05, ** *p* < 0.01. (**G**) Spontaneous action potentials recording of hUC-MSCs, iGNs, and primary neurons on MEA system (Axion Biosystems).

To explore the effects of CREB1 on mesenchymal stem cells in vitro, third-passage (P3) mesenchymal stem cells were transfected with a lentivirus overexpressing CA-CREB, and the expression of GABAergic neuronal markers was evaluated. The hMSCs overexpressing CA-CREB were stained with antibodies recognizing GABAergic neuronal markers at 14 days post-transfection. The results showed that those cells exhibited positive staining for Tuj1, Map2, NeuN, and GAD1 (Figure 3A). The statistical result showed that about 37% of hMSCs overexpressing CA-CREB displayed a neuronal phenotype (Tuj1-positive staining) (Figure S1). Levels of proteins Tuj1, Map2, and GAD1 were increased 1.5-fold, 2-fold, and 3.4-fold at 7 days post-transfection in the CA-CREB group compared with the control group transfected with a lentivirus expressing GFP alone (Figure 3D,E). Moreover, the Edu assay was performed to assess the cell proliferation of hMSCs at 4 days post-transfection. We observed that there were fewer cells in the cell cycle in the CA-CREB group compared with the control group transfected with GFP lentivirus (1.11 ± 0.93 vs. 3.78 ± 3.23) (Figure 3B,C). Notably, the muse microelectrode array (MEA) system was used to record the spontaneous action potentials of iGNs. Result showed that iGNs generated action potentials, which are similar to primary neurons, suggesting that the iGNs were electrophysiologically active (Figure 3G).

To determine whether the process of neuronal differentiation of the stem cell transition stage includes neural progenitor cells (NPCs) and neural stem cells (NSCs), we identified their markers of Sox2 and Tuj1 at 48 h after ectopic expression of CA-CREB. The results showed that the expression of the neuronal markers of Sox2 and Tuj1 increased 1.5-fold and 6.3-fold, respectively, suggesting that neural differentiation facilitated by CREB1 resulted in neuronal conversion through the stem cell transition of the NPCs/NSCs (Figure 3F).

### 3.3. RNA-seq Analysis Indicates That CREB1 Could Induce GABAergic Neuronal Expression at the Transcriptional Level

**Figure 4:**
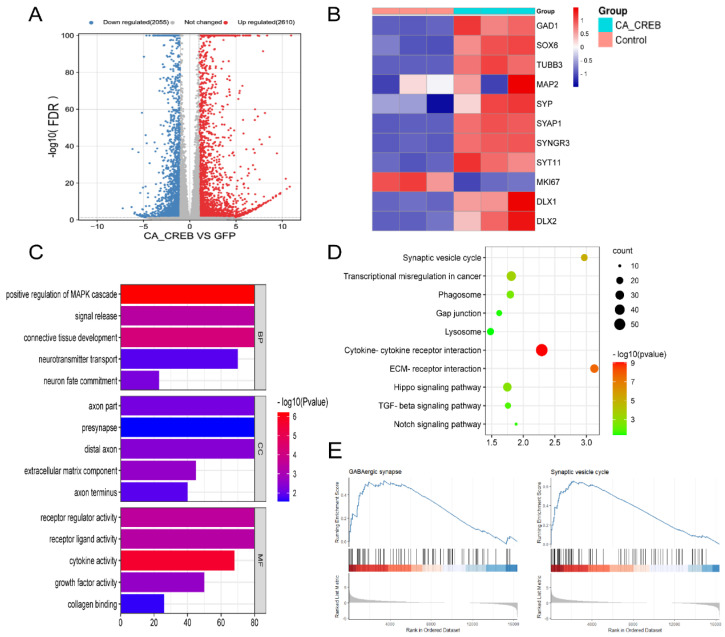
CREB1 induces GABAergic neuronal expression at the transcriptional level. GABAergic neuron cells of 7 days induced by CREB1 were carried on RNA-seq. (**A**) Results of DEGs (FDR < 0.05) are displayed in a volcano plot. (**B**) Heatmap of TFs involved in GABAergic neural development (Dlx1, Dlx2, Sox6), GABAergic neuronal markers (GAD1, Tubb3, Map2, Syp, Syap1, Syngr3, Syt11), and cell proliferation (Mki67). (**C**) All 4,665 DEGs were classified into one or more of the following three groups: biological processes (BPs), cellular components (CCs), and molecular functions (MFs). Significantly enriched GO terms are presented. (**D**) Upregulated DEGs and downregulated DEGs were analyzed separately using KEGG enrichment analysis. The top five upregulated or downregulated pathways (FDR < 0.05) are presented. (**E**) A total of 29,541 with abundance value of >1 FPKM were analyzed using gene set enrichment analysis (GSEA). The significantly enriched signaling pathways are presented.

To verify the neural differentiation of hMSCs and to explore the molecular mechanism involved in the process of differentiation, GABAergic neuron cells of 7 days induced by CREB1 were carried on RNA-seq. There were 4665 differentially expressed genes (DEGs) between the two groups (iGNs vs. hMSCs), including 2610 upregulated genes and 2055 downregulated genes (Figure 4A). The heatmap showed that the GABAergic neuronal markers of GAD1, Map2, and Syp were upregulated significantly, suggesting GABAergic neural differentiation at the transcriptional level (Figure 4B). Next, the DEGs were classified into one or more of the following three groups: biological processes (BPs), 1874 genes; cellular components (CCs), 599 genes; and molecular functions (MFs), 511 genes. GO enrichment analysis indicated that CREB1 affected a series of BPs and CCs including neurotransmitter transport, neuron fate commitment, axon part, presynapse, distal axon, and axon terminus. Furthermore, 2610 upregulated genes and 2055 downregulated genes were analyzed using KEGG analysis to investigate key signaling pathways in the differentiation process. The top five upregulated or downregulated pathways (FDR < 0.05) are shown in Figure 4D. The five upregulated pathways included synaptic vesicle cycle, transcriptional mis-regulation in cancer, phagosome, gap junction, and lysosome; the downregulated pathways included cytokine–cytokine receptor interaction, ECM–receptor interaction, Hippo, TGF-beta, and Notch signaling pathway (Figure 4D). To avoid overlooking differentially expressed genes that are not statistically significant but still have important biological significance, all expressed genes, a total of 29,541 with abundance value of >1 FPKM (fragments per kilobase of exon model per million mapped fragments), were analyzed using gene set enrichment analysis (GSEA). The results showed that the GABAergic synapse pathway and synaptic vesicle cycle pathway were significantly enriched, suggesting the formation of the GABAergic synapse upon differentiation (Figure 4E). In summary, these findings demonstrated that CREB1 could activate the hMSC differentiation to GABAergic neuron cells and promote the formation of the GABAergic synapse at the transcriptional level.

### 3.4. The Inhibition of the Notch Pathway Could Promote GABAergic Neural Differentiation In Vitro

**Figure 5:**
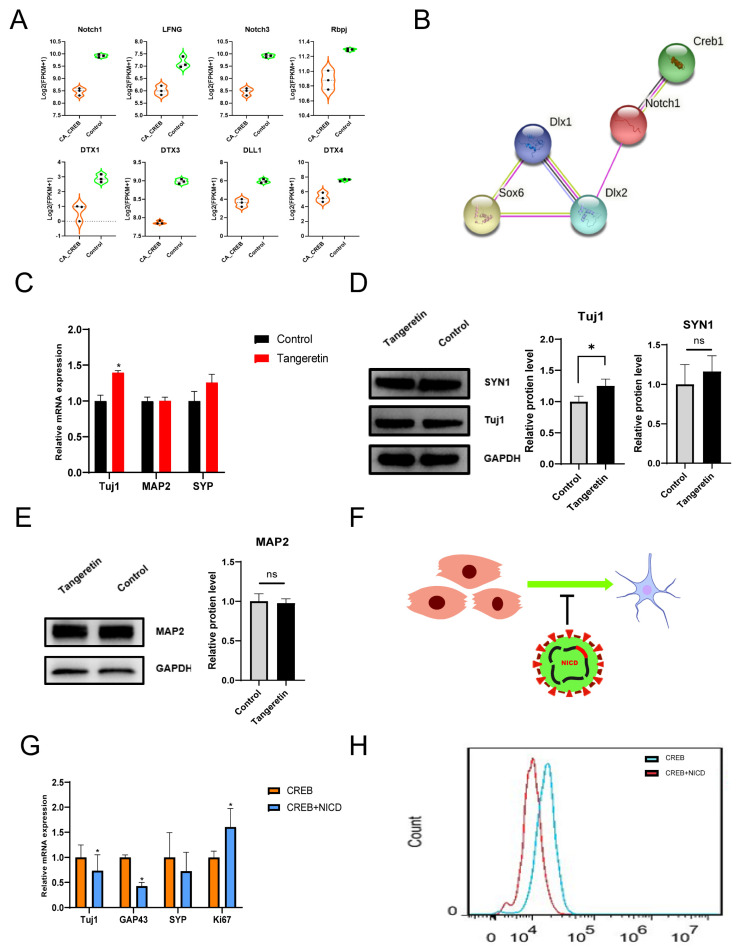
Inhibition of Notch signaling promoted GABAergic neural differentiation. (**A**) The FPKM of genes involved in the Notch pathway from RNA-seq including Notch1, Lfng, Notch3, Rbpj, Dtx1, Dtx3, Dll1, and Dtx4. (**B**) PPI network analysis of Notch1 and other TFs related to GABAergic neural differentiation (Dlx1, Dlx2, and Sox6). (**C**) RNA expression of Tuj1, Map2, and Syp in hMSCs after 7 days of treatment with or w/o tangeretin. (**D**,**E**) A Western blot image showing the expression of Tuj1, Map2, and Syn1 with or without the treatment of tangeretin and quantitative analysis. (**F**) Schematic pattern showing the inhibition of neural differentiation induced by CREB1 after overexpressing NICD. (**G**) The RNA expression of Tuj1, Gap43, Syp, and Ki67 of hUC−MSCs at 7 days after transfection with CA-CREB lentivirus and GFP (as a control) or NICD lentivirus. (**H**) Flow cytometry analysis of Tuj1 in hUC-MSCs at 7 days after transfection with CA-CREB lentivirus and GFP (as a control) or NICD lentivirus. Data are presented as mean ± SEM. Results were analyzed using *t*-test. Statistical significance: ns *p* > 0.05 * *p* < 0.05.

As shown in Figure 4D, we found that the Notch pathway is one of the significant downregulated pathways in neural differentiation, and the expression of key genes in the Notch pathway, such as Notch1 and DTX1, drops significantly in iGNs compared with hMSCs (Figure 4D and Figure 5A). Additionally, PPI network analysis showed that Notch1, the core gene in the Notch pathway, has strong interaction with CREB1 and other TFs that are related to GABAergic neural development including Dlx1, Dlx2, and Sox6, suggesting that Notch signaling inhibition participates in neural differentiation facilitated by CREB1 (Figure 5B). To validate the effect of Notch signaling on the neural differentiation of hMSCs, we added tangeretin, a small molecule inhibitor of Notch1, to the culture medium to inhibit the Notch pathway. After 7 days of cultivation, the expression of neuronal markers Tuj1, Map2, and Syp was detected using Western blot and also quantitated using RT-qPCR. The results showed that the Tuj1 RNA and protein expression increased 1.4- and 1.25-fold, respectively, in the Notch inhibition group compared with the control group (w/o tangeretin). However, the expression of Map2 and Syp did not show a significant difference. (Figure 5C–E). To further validate that notch signaling inhibition promotes neural differentiation, we overexpressed the Notch1 intracellular domain (NICD) to reverse the Notch signaling inhibition and assess the neural differentiation induced by CREB1(Figure 5F). The hUC-MSCs were co-transfected with a mixture of CA-CREB lentivirus and a lentivirus-expressing GFP (as a control) or NICD. The RNA expression of Tuj1, Gap43, Syp, and Ki67 was quantitated using RT-qPCR at 7 days post-transfection. The results showed that the RNA expression of Tuj1(*tubb3*) and Gap43 was reduced by 25% and 58%, respectively. In contrast, the RNA expression of Ki67, a marker of cell proliferation, increased 1.6-fold, indicating that NICD could enhance cell proliferation of hMSCs (Figure 5G). Additionally, flow cytometry analysis showed that less Tuj1-FITC signal was detected with coexpression of CA-CREB and NICD compared with CA-CREB alone at 7 days post-transfection (Figure 5H). This suggests that the Notch signaling inhibition is required for neural differentiation of hMSCs.

### 3.5. CREB1 Facilitates the Neuronal Maturation through Activating BRN2 Transcription In Vitro

**Figure 6:**
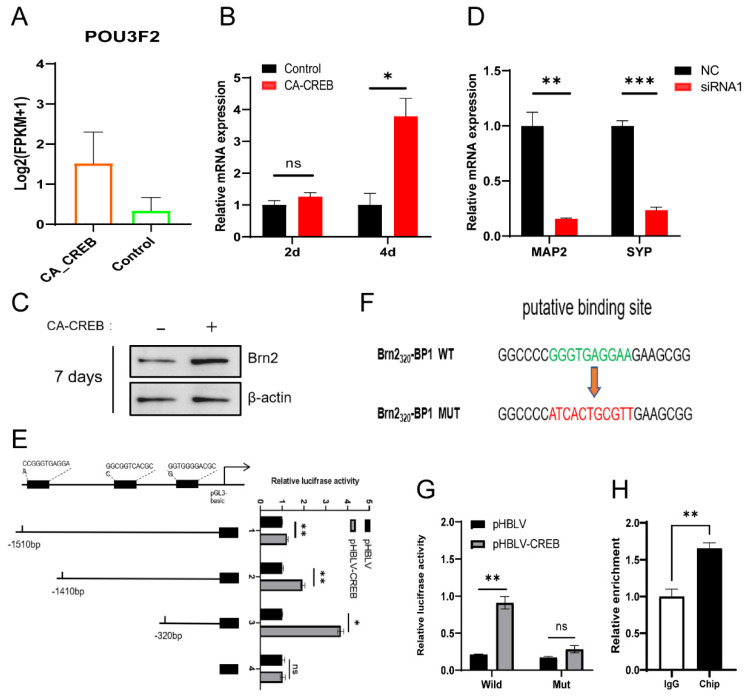
CREB1 facilitates neuronal maturation through activating BRN2 transcription. (**A**) FPKM of POU3F2 from RNA-seq result. (**B**) RNA expression of BRN2 at 2 or 4 days after transfection with lentivirus overexpressing CA-CREB or GFP (as a control). (**C**) Western blot showing the expression of BRN2 at 7 days after transfection with lentivirus overexpressing CA-CREB or GFP (as a control). (**D**) RNA expression of Map2 and Syp in hUC-MSCs at 7 days post-transfection with CA-CREB lentivirus and siRNA targeting human BRN2 or control siRNA. (**E**) Different truncated mutants of Brn2 promoter (pGL_1510_-Luc, pGL_1410_-Luc, and pGL_320_-Luc) were transfected to 293T cells with pRL-TK and pHBLV-CREB or pHBLV. At 2 days post-transfection, the 293T cells were analyzed using a luciferase assay. (**F**) Schematic pattern showing sequence of mutant BP1. (**G**) The pGL_320_-Luc or pGL_320_-Luc-M was transfected to 293T cells with pRL-TK and pHBLV-CREB or pHBLV. At 2 days post-transfection, the 293T cells were analyzed using a luciferase assay. (**H**) The 293T cells were transfected with lentivirus overexpressing CA-CREB. At 2 days post-transfection, a ChIP-qPCR assay was performed to assess the fragment enrichment of BP1. Data are presented as mean ± SEM. Results were analyzed using *t*-test. Statistical significance: * *p* < 0.05, ** *p* < 0.01, *** *p* < 0.001.

Although the Notch signaling inhibition could increase the expression of Tuj1, we did not observe any change in the expression of mature neuronal marker Map2, indicating that the GABAergic neuron-like cells originating from MSCs were arrested at a premature stage. To explore the key regulator(s), we analyzed the expression of TFs previously shown to generate mature neurons originating from human fibroblasts and astrocytes. RNA-seq data showed that the expression level of BRN2, a cortex-specific transcription factor, was significant elevated in the process of differentiation (Figure 6A). Then, we tested BRN2 expression at both the RNA level and protein level. In comparison with hMSCs, the RNA expression of BRN2 in iGNs increased after 2 days and increased by 2.79-fold at 4 days post-transfection, and the protein expression was also increased significantly after 7 days post-transfection (Figure 6B,C). To further explore the effect of BRN2 on the neuronal maturation of iGNs, we used siRNA to knock down BRN2. Four candidate siRNAs for BRN2 were transfected to hMSCs and the protein expression of BRN2 was detected at 2 days post-transfection. The result showed that siRNA1 exhibited an optimal knockdown effect and reduced the expression of BRN2 by half in hMSCs (Figure S2). Next, the hUC-MSCs were co-transfected with CA-CREB lentivirus and nontargeting siRNA (as a control) or siRNA1. We found that siRNA of BRN2 reduced the expression of Map2 and Syp by 0.16-fold and 0.23-fold at 7 days post-transfection, suggesting the crucial role of BRN2 in neuronal maturation (Figure 6D). As is shown, BRN2 was involved in the differentiation process of hMSCs; the mechanism by which CREB1 upregulates BRN2 remains unclear. To explore the underlying mechanisms, we performed bioinformatic analysis and detected three potential CREB1 binding sites (BP1, BP2, and BP3), located at 243 bp, 1397 bp, and 1497 bp upstream of the transcription initiation site of BRN2. Subsequently, we constructed three different truncated mutants, pGL_1510_-Luc, pGL_1410_-Luc, and pGL_320_-Luc (Figure 6E) and then co-transfected them with Renilla luciferase plasmid pRL-TK and plasmid overexpressing CA-CREB (pHBLV-CREB) or vector (pHBLV) to 293T cells. At 2 days post-transfection, the 293T cells were analyzed using a luciferase assay to assess the promoter-binding ability of CREB1, and the result showed that the transfection of pGL_1510_-Luc, pGL_1410_-Luc, and pGL_320_-Luc could significantly increase luciferase activity 1.23- (pGL_1510_-Luc), 1.94- (pGL_1410_-Luc), and 3.7-fold (pGL_320_-Luc) compared with the pGL3-Luc vector under the condition of CA-CREB overexpression. The pGL_320_-Luc vector was selected for subsequent experiments because it exhibited an output greater than other two truncations (pGL_1510_-Luc and pGL_1410_-Luc). To further verify that CREB1 could activate transcription by binding to the BRN2 promotor, we constructed mutants of BP1, as shown in Figure 6F, and inserted them in the pGL3 vector. The resulting plasmid was named pGL_320_-Luc-M. We then co-transfected it with pRL-TK and pHBLV-CREB or pHBLV to 293T cells. The luciferase reporter assay showed that the expression of CA-CREB did not enhance luciferase activity when pGL_320_-Luc-M was transfected to 293T cells, suggesting that mutations in the binding site lead to a reduction in CREB1 binding to the promoter (Figure 6G). Additionally, a ChIP-qPCR was performed on 293T cells at 2 days after transfection with CA-CREB overexpressing lentivirus. The result showed that fragment enrichment of the BP1 sequence increased 1.65-fold in the ChIP group compared with the IgG group, suggesting that BP1 serves as a functional binding site of CREB1 in the promoter region of BRN2; therefore, CREB1 activates BRN2 transcriptional activity and facilitates neuronal maturation (Figure 6H).

### 3.6. The Transfected hMSCs Could Differentiate to GABAergic Neuron Cells In Vivo and Promote Functional Recovery after SCI

**Figure 7:**
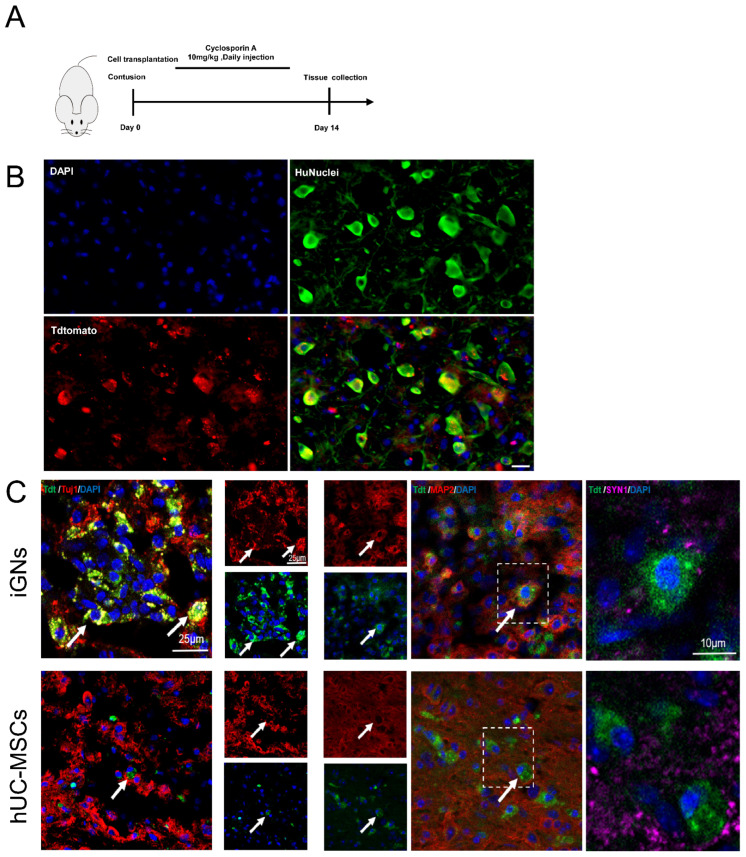
Transplanted iGNs could integrate into the local neural circuit in vivo. (**A**) Schematic pattern of cell transplantation and spinal cord tissue collection. The iGNs were injected into the lesion site immediately after SCI, and then the spinal cord tissue was obtained at 2 weeks. (**B**) Representative images showing HuNuclei immunostaining (green) in transplanted iGNs labeled with Tdtomato (red). Nuclei are labeled with DAPI (blue) in each group. Scale bars: 50 μm. (**C**) Representative images showing Tuj1, Map2, and Syn1 immunostaining at lesion site at 2 weeks after injection. Nuclei are labeled with DAPI (blue) in each group.

In order to explore the therapeutic effect of hMSC-derived GABAergic neurons on contused SCI mice, 6 × 10^5^ hUC-MSCs or iGNs were injected to the injured spinal cord locally. The spinal cord tissue was obtained at 2 weeks after injection (Figure 7A). Immunohistochemistry (IHC) analysis showed the surviving transplanted cells labeled with tdTomato (a red fluorescent protein) were detectable at the injury site until 2 weeks after transplantation, and the iGNs exhibited positive staining for neuronal markers Tuj1 and Map2, whereas a similar result was not observed in hUC-MSCs. The statistical analysis showed that about 41% of the transplanted iGNs exhibited GAD1 positive staining, indicating a GABAergic neuronal phenotype (Figure S3). Additionally, double staining with Map2 and Syn1 revealed that Map2-positive iGNs colocalized with the presynaptic marker Syn1, suggesting that transplanted iGNs integrated into the neural circuit in injured spinal cord (Figure 7C).

**Figure 8:**
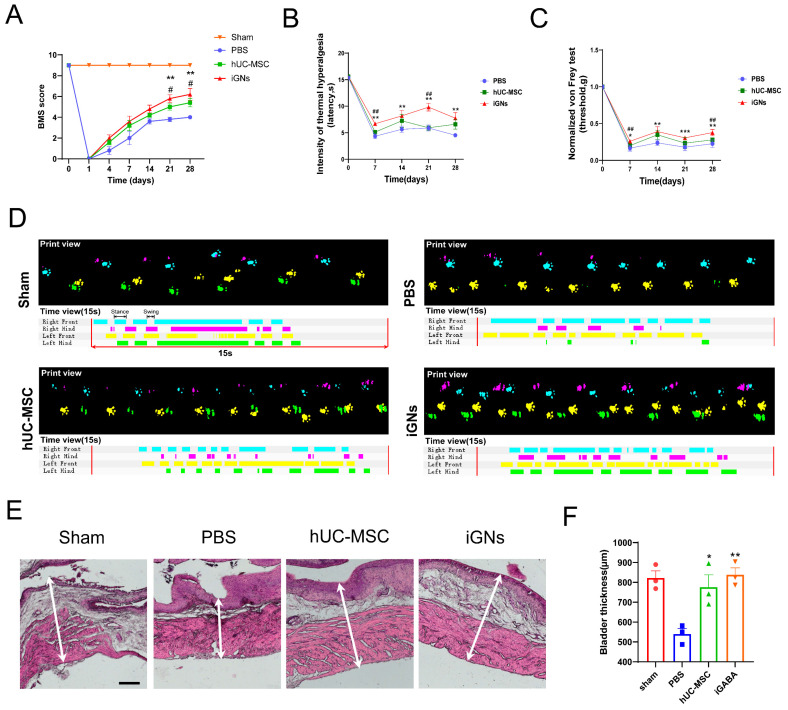
Functional recovery after iGN transplantation in contused SCI mice. (**A**–**C**) BMS scores, von Frey assay, and hot plate assay of contused SCI mice in different treatment groups after the injection of PBS, hUC-MSCs, or iGNs (sham: n = 5, PBS: n = 5, hUC-MSC: n = 5, iGN: n = 5. Data are presented as mean ± SEM. Results were analyzed using one-way ANOVA with Holm–Sidak test. Statistical significance: * *p* < 0.05, ** *p* < 0.01, *** *p* < 0.001. # *p* <0.05, ## *p* < 0.01. * denominated as comparison between the PBS group and iGN group; # denominated as comparison between the iGN group and hUC-MSC group). (**D**) Representative images of footprints after the injection of PBS, hUC-MSCs, or iGNs. The footprint diagrams of individual limbs are represented by color-coded bar graphs (bottom): the stance phase of each step is shown with solid bars and swing phase is the interval between two bars. (**E**) Representative images of HE-stained bladders in different treatment groups. Scale bars: 200 μm. (**F**) Quantification of bladder wall thickness in different treatment groups (sham: n = 3, PBS: n = 3, hUC-MSC: n = 3, iGN: n = 3). Data are presented as mean ± SEM. Results were analyzed using one-way ANOVA with Holm–Sidak test.

More importantly, we performed an animal behavior test to assess their functional recovery after transplantation. Therefore, BMS scores and gait analysis were used to assess motor function recovery in GABAergic neuron-transplanted SCI mice. The adult mice with spinal cord contusion injury were administered PBS, hUC-MSC, and iGN injections, as previously described [25]. All contused mice exhibited motor function improvement up to 4 weeks. At 4 weeks after cell injection, the SCI mice administered with iGNs showed significant improvement in their BMS score (6.2 ± 1.30 vs. 4 ± 0) compared with the PBS group. Notably, the average BMS scores of the iGN group were higher than those of the PBS group and hUC-MSC group at any time points (Figure 8A). Moreover, we assessed and quantified locomotion improvement using footprint recording analysis at the endpoint. In mice transplanted with hUC-MSCs or iGNs, coordination movement of forelimbs and hindlimbs was observed (Figure 8D). The abovementioned improvement in motor function was confirmed by the reduction in astrocytic and fibroblastic scarring in the hUC-MSC and iGN group (Figures S4 and S5). Furthermore, gait recording analysis showed that the transplantation of iGNs significantly enhanced multiple parameters of functional improvement, including regularity index (increased by 61%), average swing time (decreased by 21%), the average stand time (increased by 33%), and average maximum contact area (increased by 26%) of the hind paws compared with the PBS group (Figure S6).

To assess the ability of iGNs to alleviate neuropathic pain, the von Frey and hot plate tests were usually used to evaluate mechanical allodynia and thermal hyperalgesia in the contused mice model. From 7 days after transplantation, we observed neuropathic pain relief indicated by an increase in thermal hyperalgesia latency and a rise in mechanical pain threshold in the iGN group compared with the PBS and hUC-MSC groups (Figure 8B,C). After 4 weeks post-transplantation, the SCI mice administered iGNs showed significant improvement in both thermal hyperalgesia latency (7.74 ± 2.36 s vs. 4.52 ± 0.39 s) and mechanical pain threshold (increased by 67%), suggesting an improvement in hyperalgesia after SCI. Additionally, H&E staining showed that the bladder wall thickness was significantly higher (≈838.5 μm) in the iGN group than in the PBS group (≈540 μm) or hUC-MSC group (≈776 μm), which is similar to the sham group (≈838.5 μm) (Figure 8E,F). Taken together, this demonstrates that improved SCI recovery was achieved in the iGN group compared to the other treatment groups.

## 4. Discussion

Spinal cord injury is a severely neurological disease with complex pathological mechanisms. In SCI, the primary mechanical injury triggers a cascade of physiological responses and leads to various types of secondary damage: (1) vascular change, including hemorrhage, ischemia, and microcirculatory dysfunction; (2) the impairment of cellular integrity, including axon disruption and cell membrane damage; (3) ionic imbalance, such as increased calcium influx; (4) the imbalance of excitatory neurotransmitters, including the accumulation of extracellular glutamate, which leads to excitotoxic cell damage; (5) inflammation and edema. These responses result in the progressive cell death of neurons and glial cells and severe neurological dysfunction [5,27].

Spontaneous neurological functional recovery is rare due to inhibitory factors and the limited regenerative capacity of spinal cord neurons. The goal of intervention for SCI is to minimize secondary injury in SCI by providing neuroprotection or modulation of inflammation and rebuilding injured neural circuits through neural plasticity and axon regeneration. In the past two decades, stem cell-based therapy has demonstrated great therapeutic potential for neurological deficits in the subacute and chronic stages of SCI, which is attributed to stem cells’ ability to bridge the injury site, enhance neurite growth, and facilitate myelination [28]. Among all the reported cell types with therapeutic potential, hUC-MSCs are currently regarded as the optimal choice for cell-based therapy due to their easy accessibility, safety, low immunogenicity, and multipotency. However, recent clinical trials have demonstrated that the transplantation of hUC-MSCs has limited efficacy in alleviating neuropathic pain, despite their ability to improve motor function after SCI. Here, our findings demonstrate that CREB1 could induce hMSC differentiation to GABA neuron cells in vitro, suggesting its crucial role in GABA neural development.

CREB1 is a basic leucine zipper (bZIP) family transcription factor with a molecular size of 43 kDa, consisting of a transcriptional activity domain, a kinase-inducible domain, and a bZIP domain responsible for binding to DNA [29]. When CREB1 localizes in the nucleus, it can bind to the cAMP response element (CRE), a specialized DNA sequence TGACGTCA, and regulate the transcription of target genes such as Fos and BDNF, which is crucial in a series of biological processes in the central nervous system, including synaptic plasticity and memory formation [30,31]. However, there are few reports on its role in regulating pluripotency and neural differentiation in hMSCs. As we know, the typical transcription factors regulating the maintenance of pluripotency in ESCs, including Oct4, Nanog, and Sox2, have been proven to reprogram somatic cells into iPSCs with the potential for multilineage differentiation [32]. These transcription factors can enhance the cellular pluripotency and provide them with a broad range of differentiation fates. On the other hand, they can also lead to uncontrolled proliferation, differentiation, and even tumor formation. In our study, we observed a significant upregulation of Sox2, while the expression of Oct4 and Nanog was not detected using RT-qPCR at 2 days post-transfection, which suggests that CREB1 could enhance and maintain the pluripotency of hMSC through the Sox2-related pathway during the period of differentiation. It has been demonstrated that Sox2 tends to enhance the potential for neural lineage differentiation compared with Oct4 and Nanog, and the upregulation of Sox2 mediated by CREB1 suggests enhanced neuronal differentiation capacity in hMSCs [33].

In this study, RNA-seq data indicated the significant upregulation of Snap25 and Dnm3. Although Snap25 is not specific marker for GABAergic neurons, it is expressed in the presynaptic terminals of mature GABAergic neurons and involved in GABA release [34]. Dnm3 is a GTP-binding protein involved in the synaptic vesicular transport pathways including synaptic vesicle recycling and the maintenance of synaptic transmission. The upregulation of these two genes is a prerequisite for a functional GABAergic synapse. Additionally, we discovered that the Notch signaling pathway is a key signaling pathway participating in the neural differentiation of MSCs. Unlike previously reported pathways such as MAPK and PI3K/AKT, which promote neural differentiation in MSCs, Notch signaling is a classical pathway involved in maintaining NSC homeostasis and inhibiting neuronal differentiation [35,36]. When the Notch pathway is inhibited, it activates the pro-neuronal program at the transcription level and induces neural stem cells to differentiate into neurons or neural cells [37]. However, the precise molecular mechanism by which Notch signaling affects hMSCs is still largely unknown. On the one hand, Notch signaling inhibition plays a crucial role in regulating cell fate in various cell types, including ESCs, NSCs, and hMSCs [38]. On the other hand, CREB1-induced upregulation of Sox2 provides a prerequisite for the action of Notch signaling inhibition.

To remove hUC-MSC immunogenicity in SCI mice and promote graft survival, we immunocompromised mice by employing cyclosporine A. This approach has been validated in previous research [39]. Our data demonstrated that transplanted iGNs exhibit the expected host–graft integration and a great therapeutic effect for neuropathic pain and motor function deficiency after 2 weeks of injection, indicating potential for SCI repair. As expected, the iGN group exhibited stronger pain relief compared with the hUC-MSC group, which may be attributed to an increase in inhibitory postsynaptic potential followed by the transplantation of iGNs. Additionally, the results of behavioral assessments revealed that the iGN group exhibited comparable or even superior recovery of hindlimb motor function compared with the hUC-MSC group. Given that the transplanted iGNs did not enhance excitatory postsynaptic potentials or secrete anti-inflammatory and neurotrophic factors, recent research has reported that targeted modulation of inhibitory neurons can facilitate the integration of residual neural circuitry into the host spinal cord and improve locomotion, offering a potential explanation. However, the underlying mechanism requires further investigation to follow up.

Despite the observed therapeutic effect of iGNs on the SCI model, our study still has certain limitations. In the in vivo study, we only focused on the host integration of transplanted iGNs and the behavioral assessments on the SCI model. Other evaluation indicators, such as glial scar formation, polarization of microglia, neurotransmitter change, and electrophysiological assessments, still need further investigation for a complete and applicable evaluation. In addition, direct cell injection may cause mechanical damage to the transplanted cells. Our ongoing studies of combinatory strategies, together with hydrogel scaffolds releasing neuroprotective factors, may offer an ideal strategy for SCI repair while supporting neuron survival.

## 5. Conclusions

In this study, we observed a genetic switch, CREB1, that can activate the latent GABAergic potentiality of human mesenchymal stem cells (hMSCs) and generate GABAergic neuron-like cells originating from hMSCs. In addition, we found that GABAergic neural differentiation was enhanced, while Notch signaling was inhibited, and that BRN2 transcriptional activation plays an important role in neuronal maturation. In summary, our study offers insights into regulating the maintenance and differentiation of hMSCs, thereby presenting a new cell source of GABAergic neuron cells for SCI repair.

## Data Availability

The experimental datasets generated and/or analyzed during the current study are available from the corresponding author upon reasonable request.

## Supplementary Materials

The following supporting information can be downloaded at: https://www.mdpi.com/article/10.3390/cells13010067/s1, Figure S1. (A) The low magnification images showing Tuj1 immunostaining in hUC-MSCs at 14 days after transfection with lentivirus overexpressing CA-CREB. Scale bars, 50 μm. (B) The percentage of hUC-MSCs with a neuronal phenotype(Tuj1 postitive) in total CREB1-expressiong hUC-MSCs; Figure S2. The knockdown efficiency of different siRNAs targeting BRN2. (A,B) Four candidate siRNAs for BRN2 were transfected to hMSCs to knock down BRN2 and then the protein expression of BRN2 was detected at 2 days of post-transfection. A western blot image and analysis showing BRN2 knock down by siRNAs; Figure S3. (A) Immunostaining for GAD1 (green) of spinal cord sections in each group at 4 weeks of transplanting iGNs labeled with tdTomato. scale bar, 50 μm. (B)The percentage of hUC-MSCs with GABAergic neuronal phenotype(GAD1 postitive) in transplanted cells from 3 mice; Figure S4. (A) The representative images showing GFAP (green) immunostaining of spinal cord sections in each group at 4 weeks of post-transplantation. scale bar, 100 μm. (B) the quantification of (A) from 3 mice. Results were analyzed by ANOVA post Student-Newman-Keuls. Statistical significance: ** *p* < 0.01; Figure S5. (A) The representative images showing Fibronection (green) immunostaining of spinal cord sections in each group at 4 weeks of post-transplantation. scale bar,100 μm. (B) the quantification of (A) from 3 mice. Results were analyzed by ANOVA post Student-Newman-Keuls. Statistical significance: * *p* < 0.05, ** *p* < 0.01; Figure S6. The functional parameters from gait recording in different treatment groups. The adult mice with spinal cord contusion injury were administered d received PBS, hUC-MSCs and iGNsinjection. Four weeks after injection, the gait recording and analysis was performed; Figure S7. Schematic diagram of CREB1 facilitates GABAergic neural differentiation of hMSCs for pain alleviation and locomo-tion recovery after spinal cord injury.

## Abbreviations

SCI	spinal cord injury
hMSCs	human mesenchymal stem cells
hUC-MSCs	human umbilical cord mesenchymal stem cells
iGN	induced GABAergic neuron cell derived from hUC-MSCs
NSC	neural stem cell
NPC	neural progenitor cell
CsA	cyclosporine A
OPC	oligodendrocyte precursor cell
OEC	olfactory ensheathing cell
MSC	mesenchymal stem cell
MGE	medial ganglionic eminence
ESC-NPC	embryonic stem cell-derived neural precursor cell
iPSC	induced pluripotent stem cell
CA-CREB	constitutively active cAMP-responsive element-binding protein
DEG	differentially expressed gene
BPs	biological processes
CCs	cellular components
MFs	molecular functions
NICD	Notch1 intracellular domain
MEA	muse microelectrode array
BMS	Basso mouse scale
