# Supplementary material for: CREB1 Facilitates GABAergic Neural Differentiation of Human Mesenchymal Stem Cells through BRN2 for Pain Alleviation and Locomotion Recovery after Spinal Cord Injury
Source: Cells. 2023 Dec 28;13(1):67. doi: 10.3390/cells13010067 (PMC10778540; doi:10.3390/cells13010067)
Supplement: Supplementary file 1 [file cells-13-00067-s001.zip › cells-2667414-supplementary.pdf]

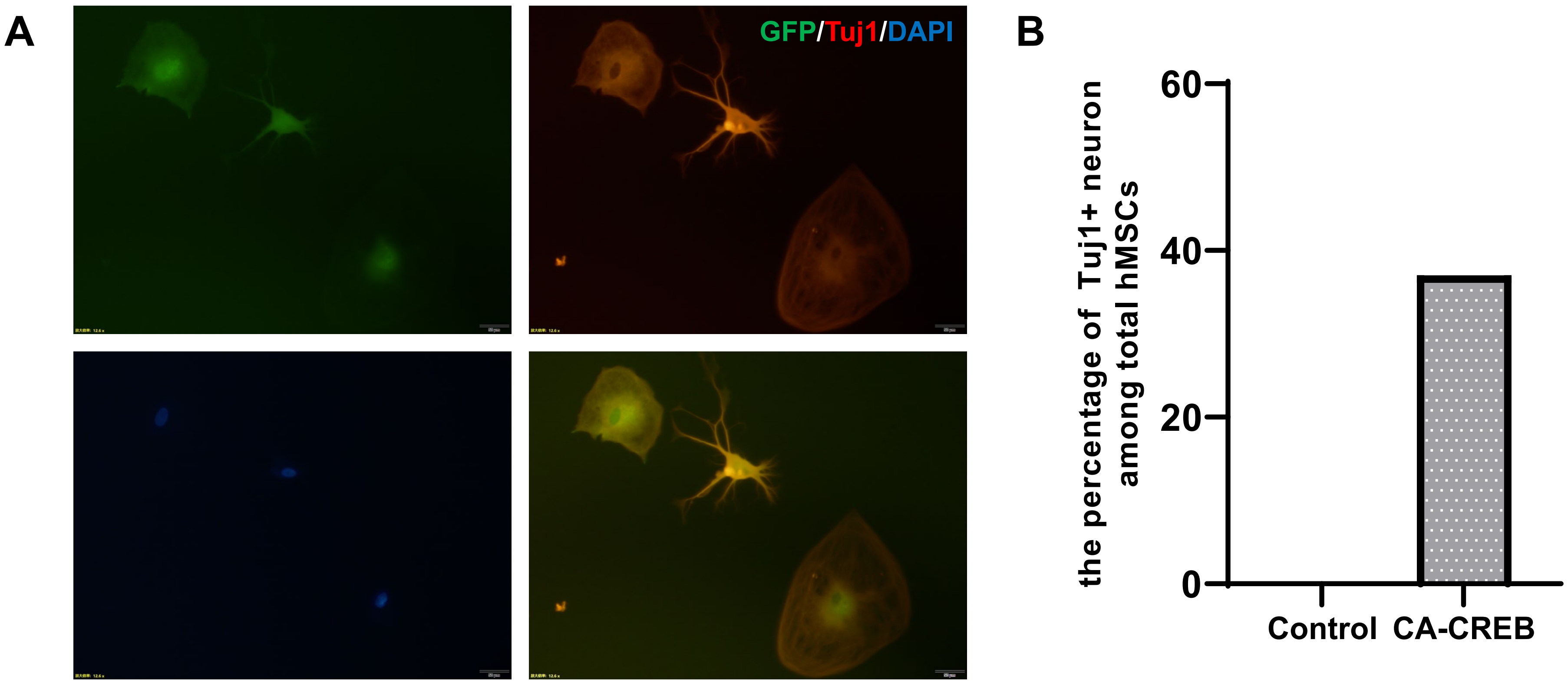


**Figure S1.** (A) The low magnification images showing Tuj1 immunostaining in hUC-MSCs at 14 days after transfection with lentivirus overexpressing CA-CREB. Scale bars, 50μm. (B) The percentage of hUC-MSCs with a neuronal phenotype(Tuj1 postitive) in total CREB1-expressiong hUC-MSCs.

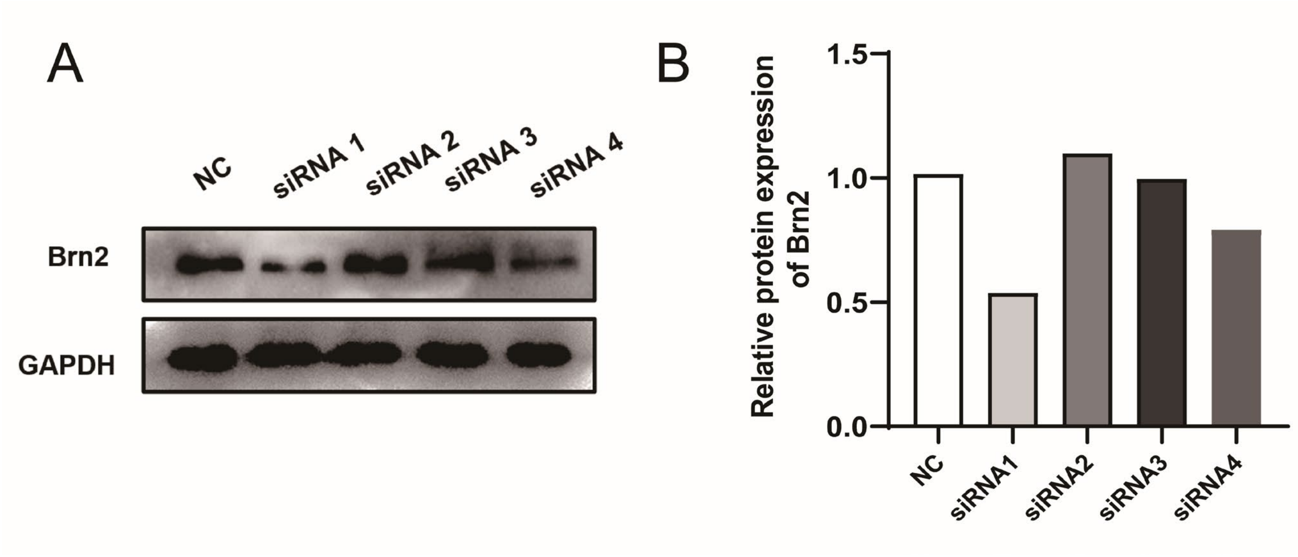


**Figure S2.** The knockdown efficiency of different siRNAs targeting BRN2. (A-B) Four candidate siRNAs for BRN2 were transfected to hMSCs to knock down BRN2 and then the protein expression of BRN2 was detected at 2 days of post-transfection. A western blot image and analysis showing BRN2 knock down by siRNAs.

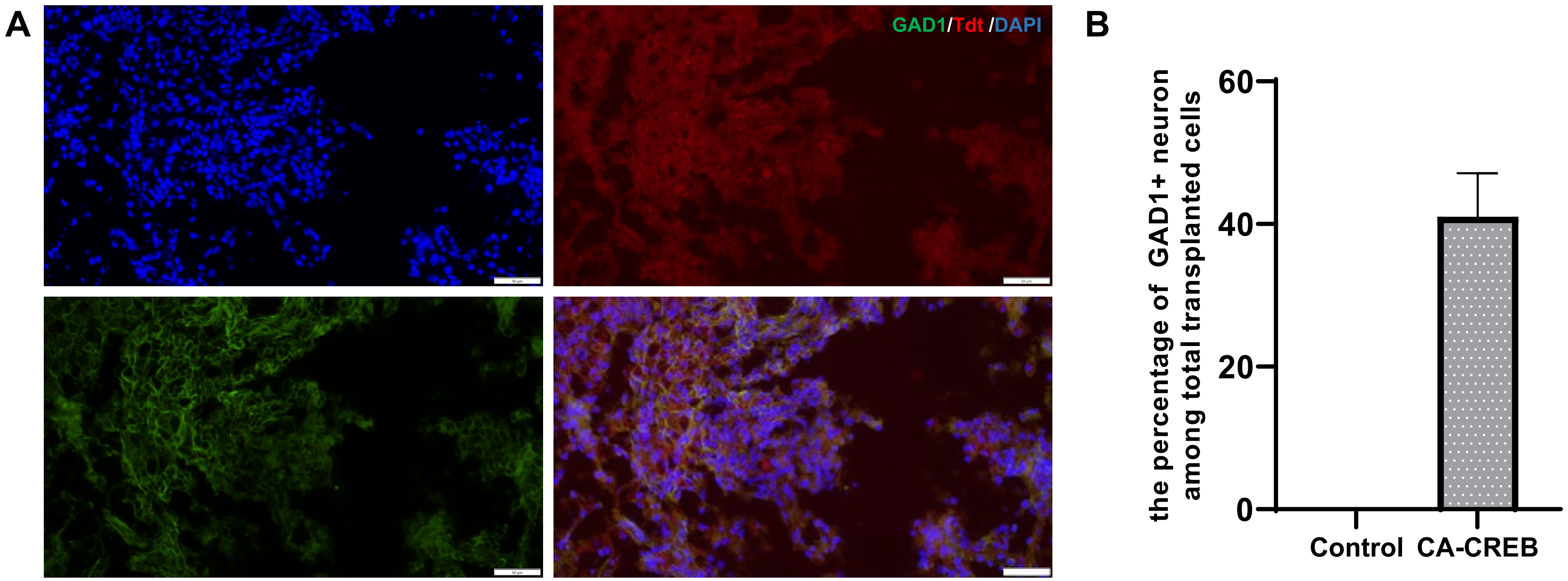


**Figure S3.** (A) Immunostaining for GAD1 (green) of spinal cord sections in each group at 4 weeks of transplanting iGNs labeled with tdTomato. scale bar,50 μm. (B) The percentage of hUC-MSCs with GABAergic neuronal phenotype(GAD1 postitive) in transplanted cells from 3 mice.

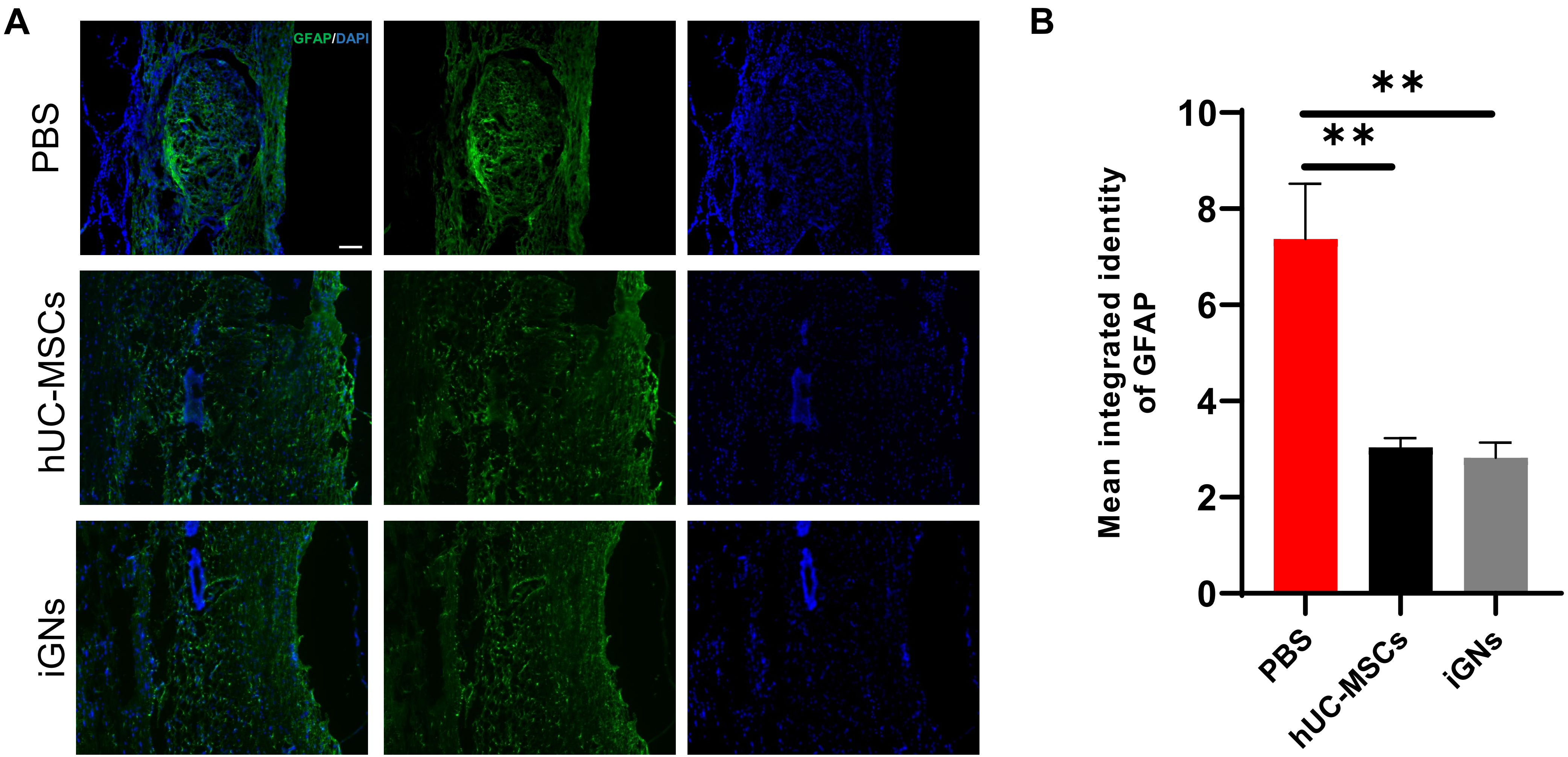


**Figure S4.** (A)The representative images showing GFAP (green) immunostaining of spinal cord sections in each group at 4 weeks of post-transplantation. scale bar,100 μm.
(B) the quantification of (A) from 3 mice. Results were analyzed by ANOVA post Student-Newman-Keuls. Statistical significance: * p <0.05, ** p <0.01, *** p <0.001

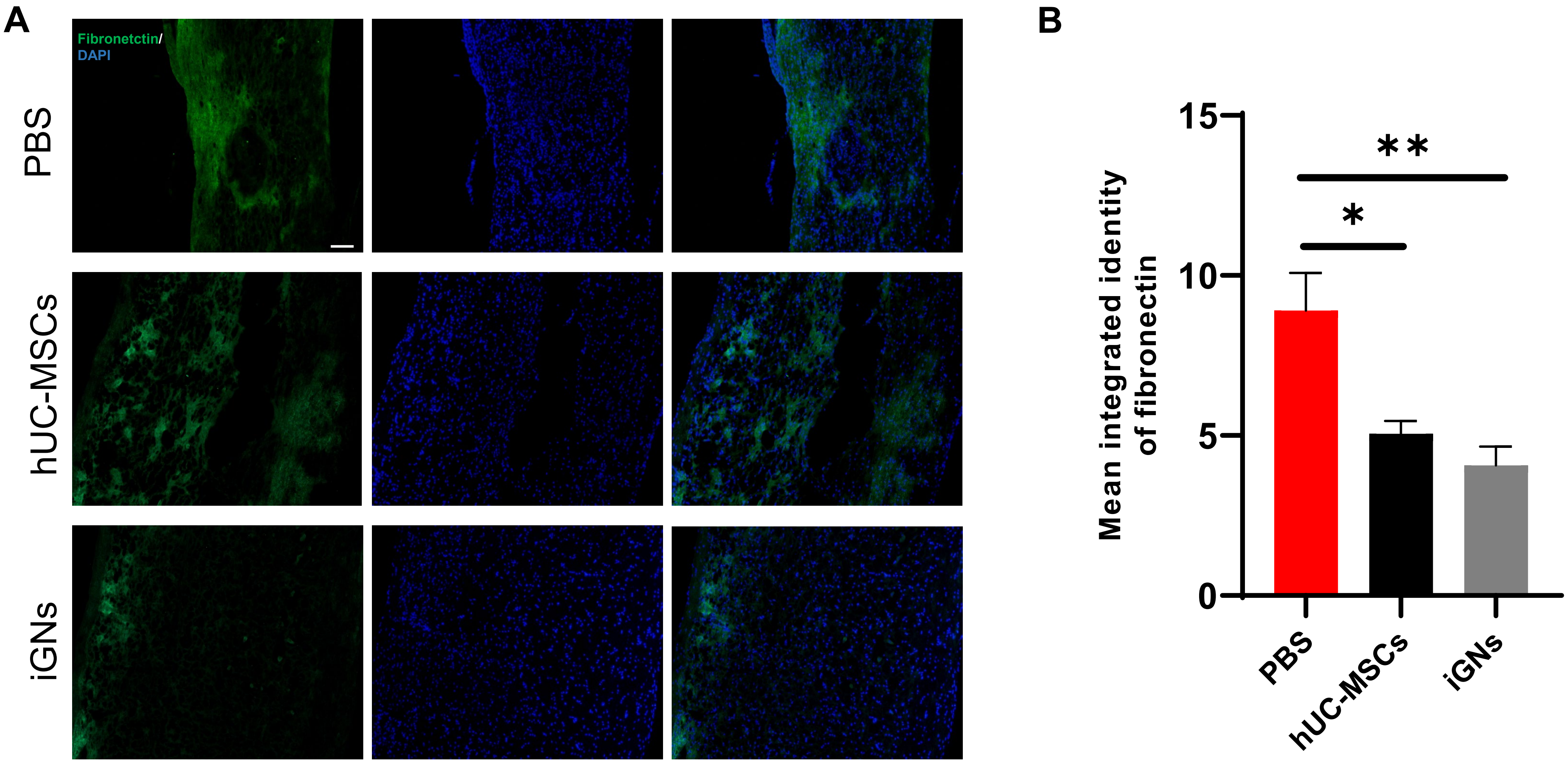


**Figure S5.** (A) The representative images showing Fibronection (green) immunostaining of spinal cord sections in each group at 4 weeks of post-transplantation. scale bar,100 µm. (B) the quantification of (A) from 3 mice. Results were analyzed by ANOVA post Student-Newman-Keuls. Statistical significance: * p <0.05, ** p <0.01, *** p <0.001.

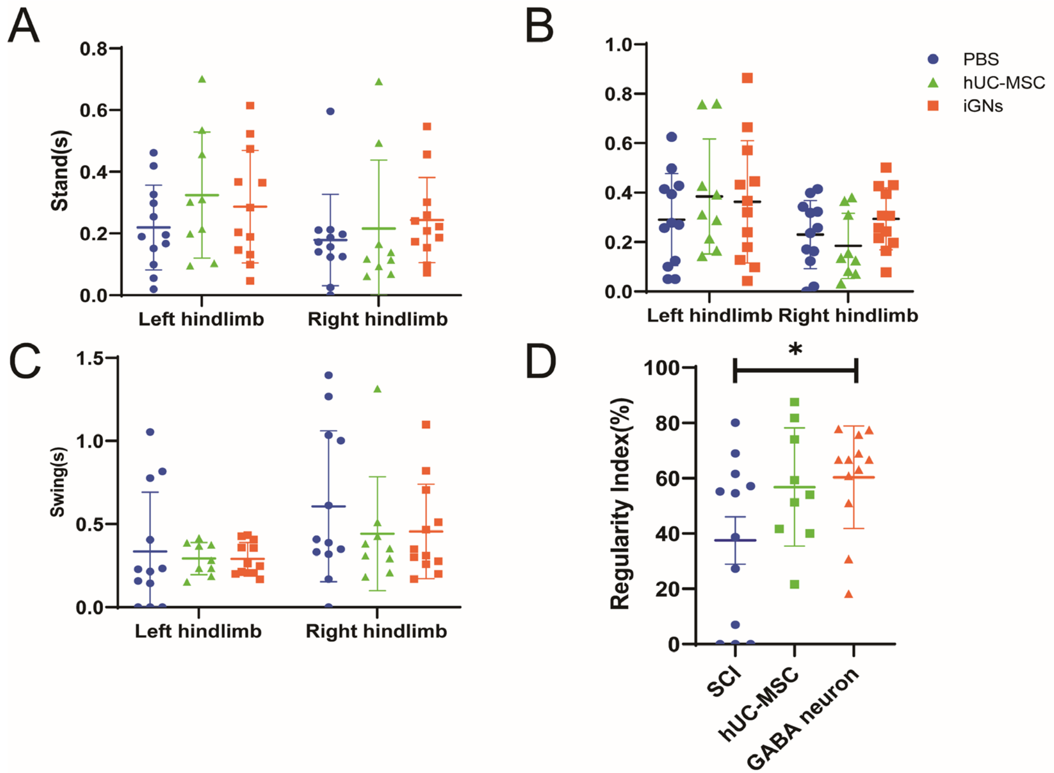


**Figure S6.** The functional parameters from gait recording in different treatment groups. The adult mice with spinal cord contusion injury were administered d received PBS, hUC-MSCs and iGNsinjection. Four weeks after injection, the gait recording and analysis was performed. (A) Stand time of left and right hindlimb in different treatment groups at 4 weeks of post-injection. (B) Max contact area of left and right hindlimb in different treatment groups at 4 weeks of post-injection. (C) Swing time of left and right hindlimb in different treatment groups at 4 weeks of post-injection. (D) Regularity index in different treatment groups at 4 weeks of post-injection. Results were analyzed by ANOVA post Student-Newman-Keuls. Statistical significance: * $p < 0.05$, ** $p < 0.01$, *** $p < 0.001$.

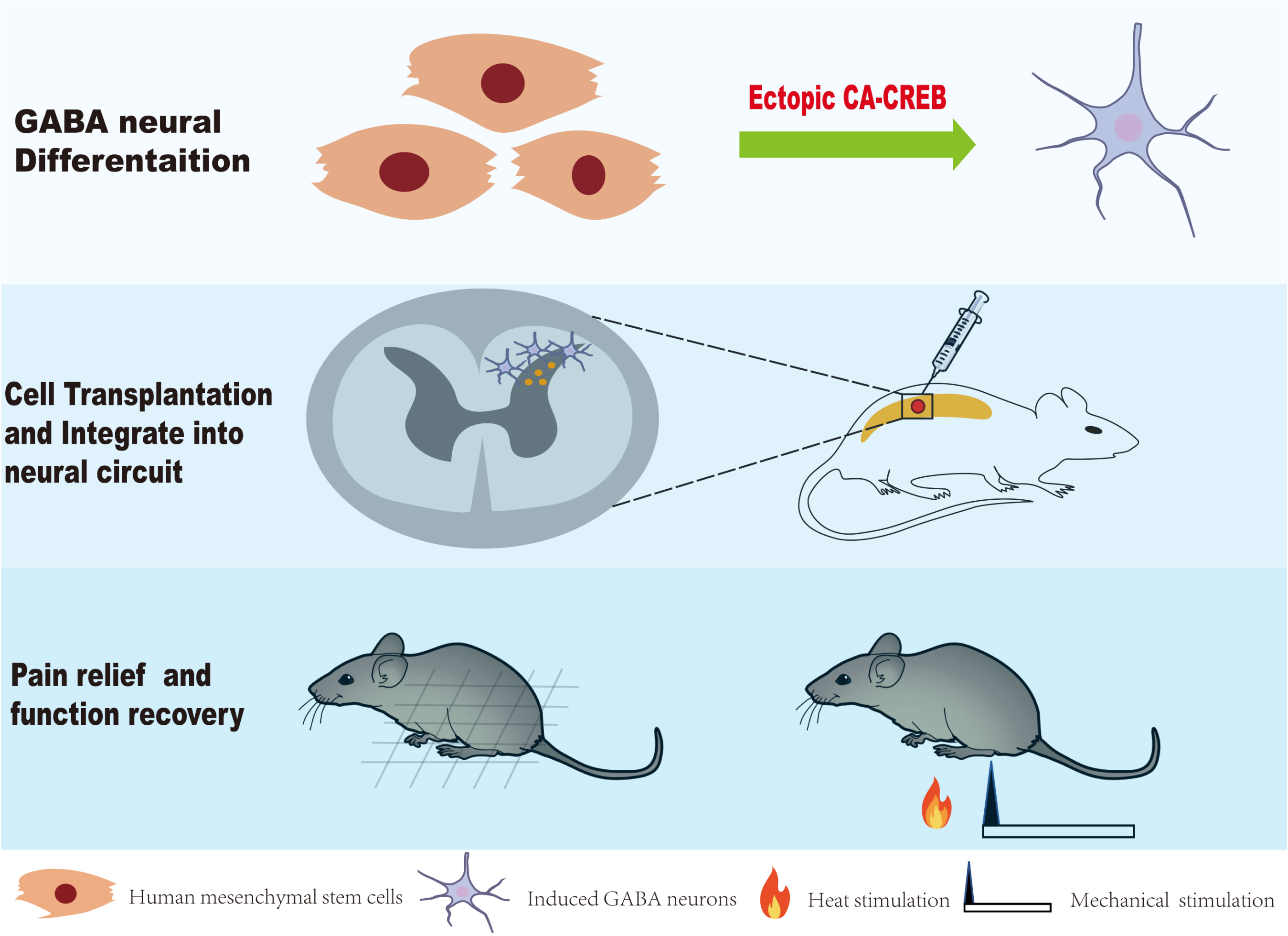


**Figure S7.** Schematic diagram of CREB1 facilitates GABAergic neural differentiation of hMSCs for pain alleviation and locomotion recovery after spinal cord injury
